# Transcatheter arterial chemoembolization combined with stereotactic body radiation therapy versus transcatheter arterial chemoembolization for hepatocellular carcinoma: a systematic review and meta-analysis

**DOI:** 10.3389/fonc.2026.1892798

**Published:** 2026-09-17

**Authors:** Dan Zeng, Xin Liu, Hao Zhang, Yaxin Zheng

**Affiliations:** 1 West China Hospital Sichuan University, Meishan Hospital, Meishan, China; 2 Department of Oncology, Meishan People’s Hospital, Meishan, China; 3 Department of Hepatobiliary and Pancreatic Surgery, Meishan People’s Hospital, Meishan, China

**Keywords:** meta-analysis, overall survival, progression-free survival, stereotactic body radiotherapy, transcatheter arterial chemoembolization

## Abstract

**Background:**

The optimal integration of locoregional therapies for unresectable hepatocellular carcinoma (HCC) remains an area of active investigation. This systematic review and meta-analysis aimed to evaluate the efficacy and safety of combining transcatheter arterial chemoembolization (TACE) with stereotactic body radiotherapy (SBRT) compared to TACE.

**Methods:**

A comprehensive literature search was conducted across five databases up to March 1, 2026. Eligible studies compared TACE combined with SBRT versus TACE (with or without concurrent systemic therapy) for unresectable HCC. The primary outcomes were overall survival (OS) and progression-free survival (PFS), with treatment-related adverse events (CTCAE grade ≥3) as a key safety outcome. Pooled hazard ratios (HR) and risk ratios (RR) with 95% confidence intervals (CI) were calculated using random-effects models. Heterogeneity was assessed using Cochran’s Q test and the *I²* statistic. Subgroup analyses (by study design, concurrent systemic therapy, and HR adjustment) and sensitivity analyses were performed.

**Results:**

Nine studies involving 828 patients were included. The TACE-SBRT combination significantly improved OS (HR = 0.48, 95% CI: 0.35–0.65, *P* < 0.001; I² = 13%) and PFS (HR = 0.45, 95% CI: 0.32–0.65, *P* < 0.001; I² = 54%) compared to TACE. There was no statistically significant difference in the incidence of grade ≥3 adverse events between the two groups (RR = 1.27, 95% CI: 0.87–1.86, *P* = 0.21). Sensitivity analyses confirmed the robustness of the findings.

**Conclusion:**

In patients with unresectable HCC, the combination of TACE and SBRT appears to confer a survival advantage over TACE, without significantly increasing the risk of severe adverse events.

**Systematic Review Registration:**

https://www.crd.york.ac.uk/PROSPERO/view/CRD420261350374, identifier CRD 420261350374.

## Introduction

Hepatocellular carcinoma (HCC) remains a leading cause of global cancer-related mortality, with its high incidence and poor prognosis sustained by frequent late-stage diagnosis, which precludes curative surgical intervention (1, 2). For patients with unresectable HCC classified under the Barcelona Clinic Liver Cancer (BCLC) intermediate stage, transcatheter arterial chemoembolization (TACE) is the established standard of care (3). However, the therapeutic landscape of unresectable HCC has evolved substantially in recent years, with systemic therapies—including tyrosine kinase inhibitors (TKIs) and immune checkpoint inhibitors (ICIs)—increasingly integrated alongside locoregional treatments (4). Despite this shift, TACE remains a cornerstone for many patients, either as monotherapy or in combination with systemic agents.​ TACE induces tumor necrosis through dual mechanisms comprising arterial embolization to induce ischemia and localized delivery of chemotherapy (5). However, the efficacy of TACE, whether as monotherapy or even when combined with systemic therapies, is often limited by the common occurrence of local recurrence and distant metastasis primarily results from technical challenges such as incomplete embolization (6) and vascular recanalization (7), coupled with the biological challenge of residual tumor cells that often persist, especially in the peri-tumoral region (8). This is further compounded by the hypoxia-induced upregulation of pro-angiogenic factors like vascular endothelial growth factor (VEGF), which may paradoxically promote tumor progression (9, 10). For large tumors, single-session TACE frequently yields suboptimal local control (11), while repeated procedures can impair hepatic reserve (12), diminishing patient tolerance and quality of life. Consequently, developing more effective combinatorial strategies is a critical imperative in managing unresectable HCC (13).

Stereotactic Body Radiation Therapy (SBRT), as a high-precision, hypofractionated radiotherapy technique, can deliver ablative radiation doses to intrahepatic tumor targets while minimizing damage to surrounding normal liver tissue (14). Its integration with TACE presents a compelling therapeutic synergy, as SBRT can target and eradicate the residual, often hypoxic, cancer cells that survive after TACE (15), especially in poorly vascularized regions (16). Clinical observations support this rationale. For instance, sequential SBRT following an incomplete response to TACE has been shown to improve local control and survival outcomes (17). Preliminary comparative studies also suggest a potential survival advantage for the TACE-SBRT combination over other non-surgical therapies in select patient cohorts (18). Furthermore, the advent of systemic therapies has not diminished but rather complemented the role of SBRT, as preclinical and early clinical data indicate that SBRT may synergize with TKIs and ICIs through immunomodulatory effects and vascular normalization (19, 20).

Despite the potential shown by combination therapy, current evidence primarily stems from single-arm or small-sample controlled studies with inconsistent results. As shown in the RCT conducted by Comito et al. (21), the TACE-SBRT combination significantly prolongs progression-free survival (PFS) versus TACE monotherapy. Wong et al. (22) also reported superior tumor control with this approach, collectively suggesting a therapeutic benefit for the combined modality. More recently, randomized trials such as Duan et al. (23) have extended this comparison to TACE-SBRT plus TKIs versus TACE plus TKIs, reflecting the growing integration of systemic therapy into the TACE-SBRT paradigm.​ However, a comprehensive and quantitative synthesis of the efficacy and safety profile of this combined approach is lacking. Therefore, to provide higher-level evidence for clinical decision-making, this systematic review and meta-analysis aim to synthesize existing comparative data to evaluate the survival benefits and safety of combining TACE with SBRT versus TACE (with or without concurrent systemic therapy)​ in patients with unresectable HCC.

## Methods

### Study design and registration

This systematic review and meta-analysis was conducted in accordance with the Preferred Reporting Items for Systematic Reviews and Meta-Analyses (PRISMA) guidelines. The study protocol was prospectively registered on the International Prospective Register of Systematic Reviews (PROSPERO; registration number CRD 420261350374). All analytical procedures strictly followed the pre-specified protocol without major deviations.

### Literature search strategy

The following electronic databases were systematically searched from their inception dates up to March 1, 2026: PubMed, MEDLINE, Embase, Cochrane Library, and Web of Science. The search strategy was structured based on the PICOS framework (**Supplementary Table S1**) and employed a combination of subject headings (MeSH/Emtree) and free-text terms. Core search terms included: “hepatocellular carcinoma”, “HCC”, “ transcatheter arterial chemoembolization”, “TACE”, “stereotactic body radiotherapy”, “SBRT”, “stereotactic ablative radiotherapy”, “SABR”, etc. These terms were combined using Boolean operators (AND, OR). Additionally, the reference lists of relevant reviews and included studies were manually searched to identify potentially eligible publications. The specific search strategy for each database is detailed in **Supplementary Table S2**. Two reviewers independently screened each identified study according to pre-defined criteria. Any disagreements regarding study inclusion were resolved through discussion and consultation with a third, experienced reviewer.

### Eligibility criteria

#### Inclusion criteria

Studies were included if they met the following criteria:

- Population:​ Adult patients (≥18 years) with HCC confirmed by pathology or clinical imaging criteria, who were not candidates for curative-intent treatments (liver transplantation, surgical resection, or thermal ablation). Concurrent systemic therapy (e.g., tyrosine kinase inhibitors [TKIs], immune checkpoint inhibitors [ICIs], or their combinations) was not an exclusion, provided that the systemic regimen was administered equivalently across both the experimental and control groups.
- Intervention:​ The experimental group received TACE combined with SBRT. Both sequential and concurrent approaches were eligible. Explicit reporting of the TACE regimen (chemotherapeutic agents, dosages, embolic materials, and administration frequency) and SBRT parameters (radiation dose, fractionation schedule, and target volume) was required, although specific parameter thresholds were not restricted. Concurrent or adjuvant systemic therapy was permitted as long as it was not the primary variable differentiating the two study arms.
- Comparator:​ The control group received TACE alone or TACE plus the same systemic therapy as the experimental group. The TACE regimen was required to be consistent with or comparable to that used in the experimental group.
- Outcomes:​ Studies were required to report at least one of the following outcomes: overall survival (OS), PFS or treatment-related adverse events (TRAEs) graded according to the Common Terminology Criteria for Adverse Events (CTCAE).

#### Exclusion criteria

Studies were excluded if they met any of the following criteria:

- Case reports, case series, narrative reviews, animal studies, *in vitro* experiments, and conference abstracts (unless full data were provided or could be obtained by contacting the authors).
- Studies involving patients with other concurrent primary malignancies, severe hepatic or renal insufficiency, uncorrectable coagulation disorders, uncontrolled severe infections, or patients deemed unable to tolerate TACE or SBRT.
- Studies with incomplete or unextractable outcome data.
- Duplicate publications or overlapping cohorts; for such cases, only the study with the most comprehensive data and the largest sample size was included.

### Literature screening and data extraction

Literature screening and data extraction were conducted independently by two investigators. Any discrepancies were resolved by consensus or by consulting a third, senior reviewer. The extracted data encompassed: (1) study characteristics (first author, publication year, country, study design, sample size); (2) patient baseline characteristics (age, liver function, tumor size and number); (3) intervention details for both the TACE-SBRT combination and TACE groups; (4) follow-up duration; and (5) outcome data for OS, PFS, and TRAEs.

### Risk of bias assessment

Two reviewers independently assessed the risk of bias in the included RCTs using the Cochrane Risk of Bias tool version 2 (ROB2). Each study was judged across five domains, with results categorized as “low risk,” “some concerns,” or “high risk.” For non-RCTs, the Risk of Bias In Non-randomized Studies - of Interventions (ROBINS-I) tool was used, assessing risk of bias across seven core domains and classifying it as “low risk,” “moderate risk,” “serious risk,” or “critical risk.” Disagreements during the assessment process were resolved through discussion involving a third reviewer.

### Certainty of evidence

The certainty of evidence for each outcome was independently assessed by two reviewers using the Grading of Recommendations Assessment, Development and Evaluation (GRADE) framework. Evidence was evaluated across five domains: risk of bias, inconsistency, imprecision, indirectness, and publication bias. The final certainty was subsequently categorized as high, moderate, low, or very low. Furthermore, the overall methodological quality of this systematic review was evaluated with the A Measurement Tool to Assess Systematic Reviews (AMSTAR) 2 checklist.

### Statistical analysis

Statistical analyses were performed using the Cochrane Review Manager (RevMan, Version 5.4) software. For time-to-event survival outcomes (OS, PFS), the hazard ratio (HR) with its 95% confidence interval (CI) was used as the effect measure. The HR and 95% CI reported in the studies were extracted directly; adjusted HR and their 95% CI were prioritized if available, and pooled using the inverse-variance method. If HRs were not directly reported, the method described by Parmar et al. (24) was used to extract HR and 95% CI from survival curves. For dichotomous outcomes (adverse events), the risk ratio (RR) with a 95% CI was calculated.

Study heterogeneity was assessed using Cochran’s Q test and the I² statistic. Heterogeneity was considered low if the Q test P-value was >0.10 and the I² value was <50%; conversely, a P-value ≤0.10 or an I² value ≥50% indicated substantial heterogeneity. Given the potential for clinical and methodological heterogeneity among the included studies, a random-effects model was used to pool effect estimates. Pre-specified subgroup analyses were conducted to explore sources of heterogeneity, categorizing studies by study design (RCTs vs. N-RCTs), statistical adjustment of HR (adjusted vs. unadjusted), and concurrent systemic therapy (present vs. absent). Sensitivity analysis using the leave-one-out method was employed to assess the robustness of the results. Publication bias was evaluated visually with funnel plots and statistically with Egger’s test.

## Results

### Literature search

Initial systematic searches across five databases up to March 1, 2026, yielded 1100 records. After removing duplicates and screening titles, abstracts, and full texts, nine studies (21–23, 25–30) met the eligibility criteria and were included in the meta-analysis. The detailed selection process is summarized in the PRISMA flow diagram (**Figure 1**).

**Figure 1:**
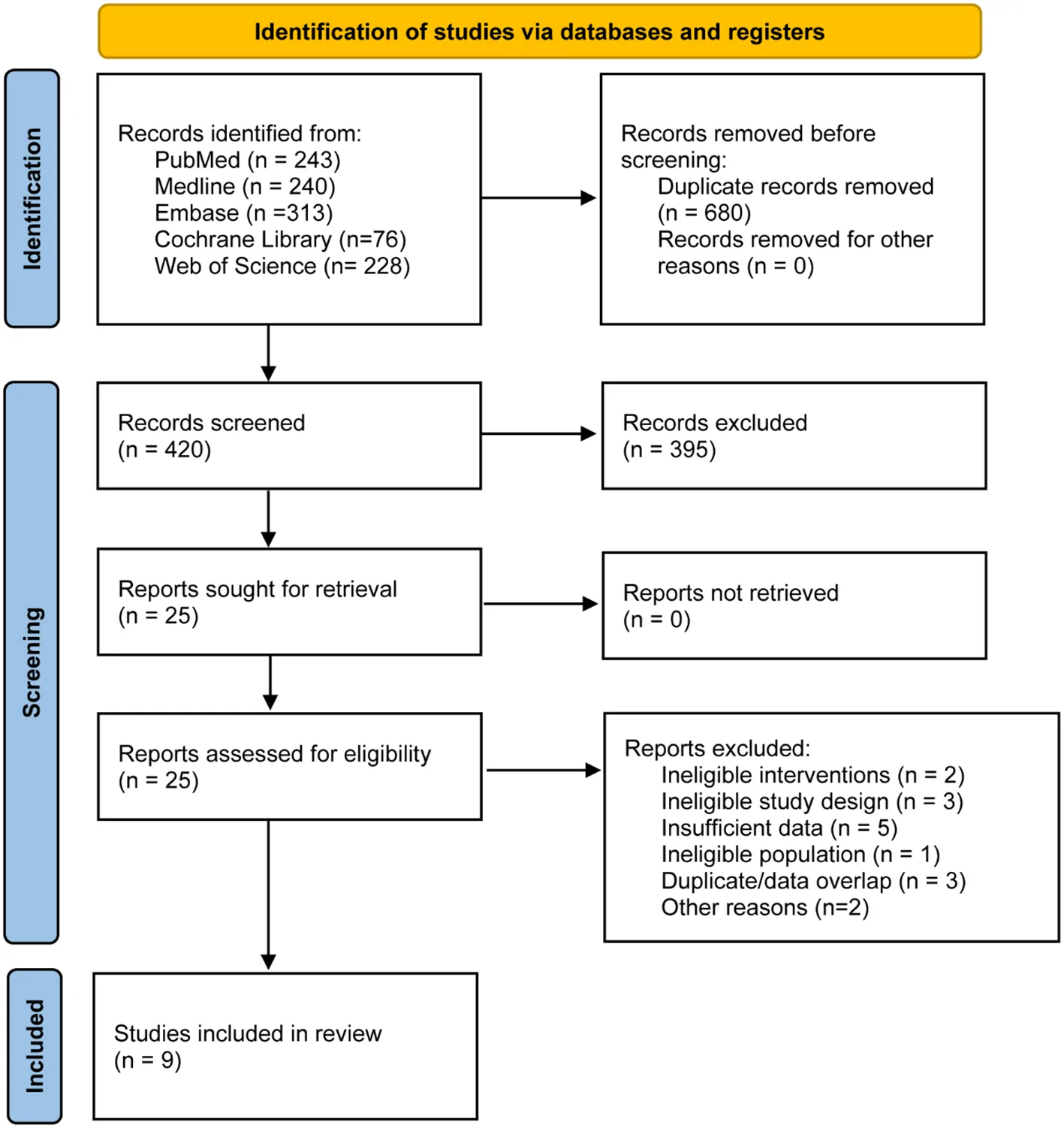
Flowchart of literature screening (PRISMA flow diagram).

### Characteristics of included studies

The nine included studies comprised three RCTs (21, 23, 28) and six N-RCTs (22, 25–27, 29, 30), published between 2013 and 2025. These studies originated from five countries: four were conducted in China (22, 23, 29, 30), two in South Korea (26, 27), and one each in Japan (25), Egypt (28), and Italy (21). In total, 828 patients with unresectable HCC were analyzed, with 358 receiving TACE combined with SBRT and 470 receiving TACE. Baseline characteristics, including age, sex, Child-Pugh grade, tumor size, and number, were comparable between the two groups (**Table 1**). In the combination group, TACE was primarily performed using epirubicin or cisplatin with lipiodol. SBRT was delivered in 3 to 10 fractions, with a median biological effective dose (BED) ranging from 60 to 100 Gy (**Supplementary Table S3**).

**Table 1:** Baseline characteristics of the included studies.

Study	Country	Study type	Number of Patients		Gender (M/F)		Age (Year)		Tumor size (cm)		Tumor number (1/2/≥3)		Child status (A/B)		Median follow-up time (months)
			SBRT+TACE	TACE	SBRT+TACE	TACE	SBRT+TACE	TACE	SBRT+ TACE	TACE	SBRT+TACE	TACE	SBRT+TACE	TACE	
Honda (2013) (23)	Japan	N-RCT	30	38	19/11	15/23	70(49-90)	73(48-92)	1.6(1.0-3.0)	2.1(0.6-3.0)	NA	NA	24/6	31/7	12.3/30.2
Paik (2016) (27)	Korea	N-RCT	37	70	29/8	49/21	59(42–74)	59(24–84)	3.4(0.8–10)	3.8(0.5–10)	NA	NA	NA	NA	35
Jun (2018) (26)	Korea	N-RCT (PSM)	85	85	65/20	64/21	62.6 ± 10.0	62.8 ± 10.6	2.23 ± 1.17	2.29 ± 1.17	55/20/10	51/23/11	71/14	74/11	NA
Wong (2019) (22)	China	N-RCT (PSM)	49	98	42/7	80/18	61(28–87)	65(20–90)	9.5(4–23.6)	10.1(1.8–22.4)	27/7/15	56/13/29	46/3	85/13	13
Comito (2022) (21)	Italy	RCT	21	19	15/6	15/4	NA	NA	2.5		14/2/5	9/7/3	17/4	14/5	20
Xiang (2022) (29)	China	N-RCT	31	45	23/8	39/6	NA	NA	4.3 (1.6-6)	4.8(1.3-12)	0/20/11	0/31/14	31/0	44/1	10/11
Saad (2024) (28)	Egypt	RCT	20	22	15/5	16/6	58.1 ± 8	60.2 ± 8.3	4.9 ± 1.1	4.4 ± 0.9	12/7/1	12/10/0	18/2	21/1	20
Duan (2024) (23)	China	RCT	45	45	NA	NA	55(21-74)	54(31-67)	NA	NA	NA	NA	NA	NA	14.5
Chen (2025) (30)	China	N-RCT	40	48	32/8	36/12	60.3 ± 7.9	60.1 ± 8.0	5.2 ± 1.1	4.7 ± 0.9	28/10/2	26/22/0	40/0	48/0	20

SBRT, stereotactic body radiotherapy; TACE, arterial chemoembolization; PSM, propensity score matched; NA, Not available; RCT, Randomized controlled trial.

### Risk of bias assessment

For the three RCTs, assessed using the RoB 2 tool (31), the study by Saad et al. (28) and Comito et al. (21) was judged to have a low risk of bias across all domains. The study by Duan et al. (23) raised some concerns due to unclear reporting of the randomization process and outcome measurement (**Supplementary Figure S1**).

For the six non-randomized studies, assessed using the ROBINS-I tool (32), the primary risk of bias arose from the control of confounding factors. Two studies were rated as “low risk” because they employed propensity score matching (PSM) to adequately control for key confounding factors such as tumor burden and liver function in their design and analysis. Four studies were rated as “moderate risk” due to potential unmeasured confounding factors (**Supplementary Table S4**).

### Meta-analysis of survival outcomes

#### Overall survival

Data from seven studies were pooled. The pooled analysis illustrated that the combination of TACE and SBRT was associated with a significantly improved OS compared to TACE (HR = 0.48, 95% CI: 0.35–0.65, P < 0.001), with no significant heterogeneity (I² = 13%, P = 0.33; **Figure 2**). This benefit was consistent across pre-specified subgroups based on study design (**Supplementary Figure S2**), statistical adjustment (**Supplementary Figure S3**) and concurrent systemic therapy (**Supplementary Figure S4**).

**Figure 2:**
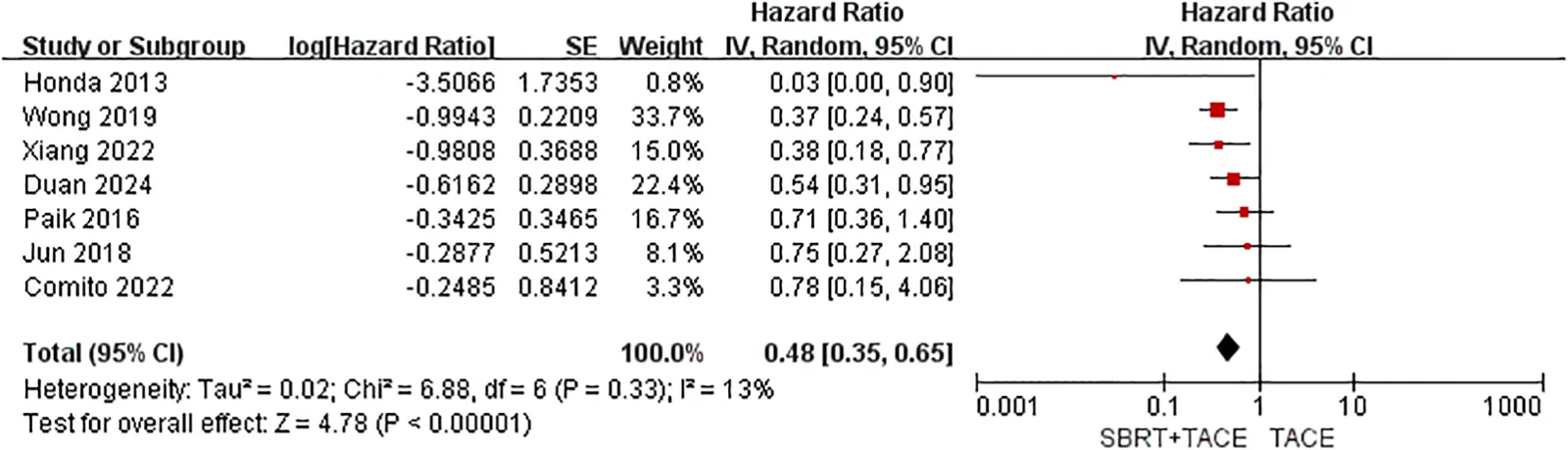
Forest plot for overall survival (TACE+SBRT vs. TACE).

#### Progression-free survival

Data from eight studies were pooled. The pooled analysis indicated that combination therapy significantly prolonged PFS compared to TACE (HR = 0.45, 95% CI: 0.32–0.65, P < 0.001), with moderate heterogeneity (I² = 54%, P = 0.03; **Figure 3**). Subgroup analyses confirmed a consistent PFS advantage across all strata, including study design (**Supplementary Figure S5**), adjustment status (**Supplementary Figure S6**) and concurrent systemic therapy (**Supplementary Figure S7**).

**Figure 3:**
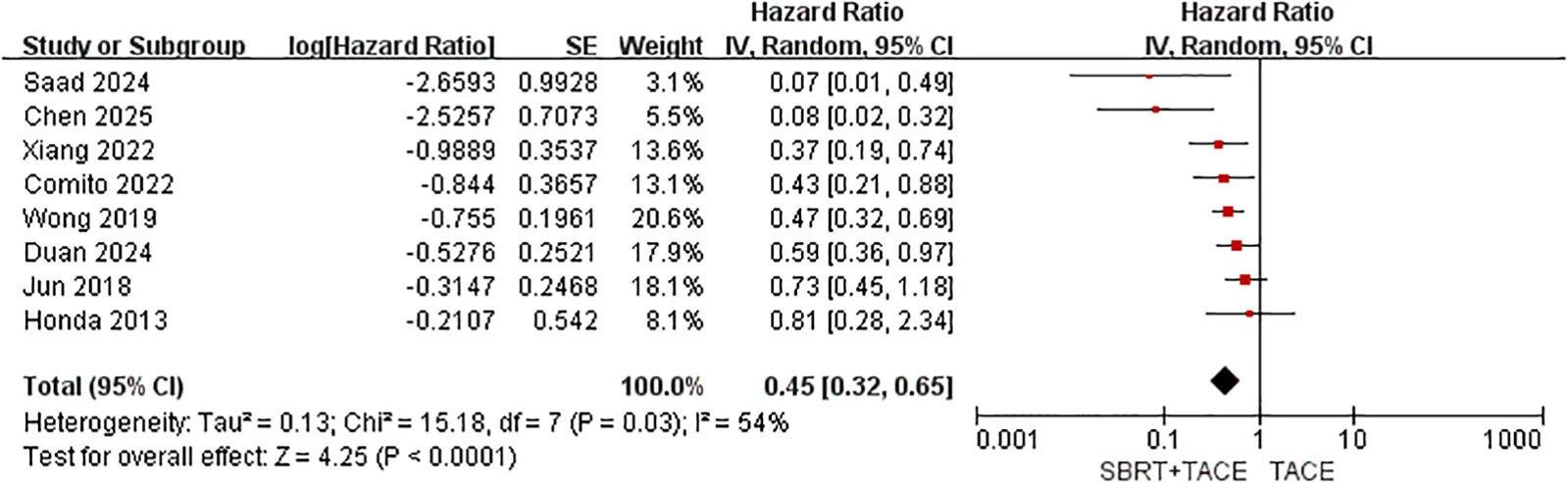
Forest plot for progression-free survival (TACE+SBRT vs. TACE).

#### Treatment-related adverse events

Eight studies reported the overall treatment-related adverse events (CTCAE ≥ grade 3). The pooled analysis showed no significant difference in the incidence of overall adverse events between the combination therapy and the TACE (RR = 1.27, 95% CI: 0.87-1.86, P = 0.21), with no significant heterogeneity (I² = 0%, P = 0.49; **Figure 4**). No cases of radiation-induced liver injury of grade ≥3 were reported in the included studies.

**Figure 4:**
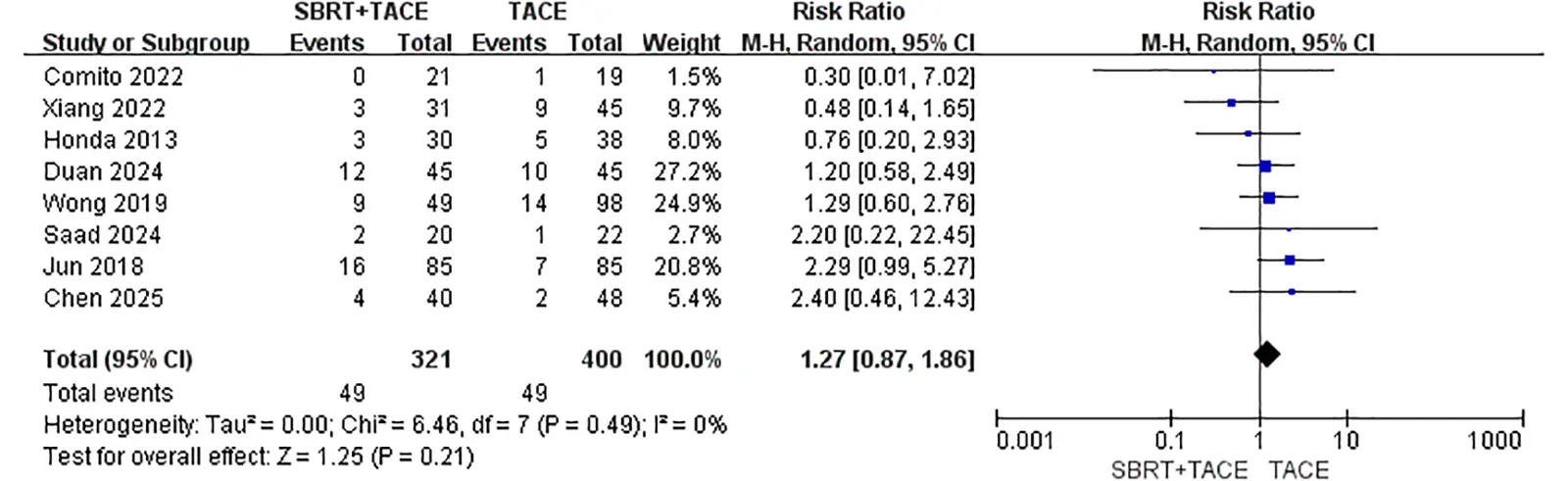
Forest plot for treatment-related adverse events (TACE+SBRT vs. TACE).

### Sensitivity analysis and certainty of evidence

Leave-one-out sensitivity analysis confirmed that the pooled estimates for OS, PFS, and adverse events were robust and not unduly influenced by any single study (**Supplementary Figure S8**).

According to the GRADE framework, the certainty of evidence for OS, PFS and treatment-related adverse events was rated as moderate, downgraded due to the risk of confounding in non-randomized studies (**Supplementary Table S5**). The overall methodological quality of this review, assessed using AMSTAR 2, was high, with all critical domains met (**Supplementary Table S6**).

### Publication bias

Funnel plots and Egger’s test were employed to assess potential publication bias across three primary endpoints: OS, PFS, and treatment-related adverse events. A visual examination of the funnel plots for these outcomes demonstrated a symmetrical distribution of the included studies, with no apparent asymmetry indicative of small-study effects. Quantitatively, the P-values obtained from Egger’s regression test for OS, PFS, and grade ≥ 3 adverse events were 0.67, 0.07, and 0.54, respectively, all of which exceed the 0.05 significance threshold (**Supplementary Figure S9**).

## Discussion

This systematic review and meta-analysis synthesized data from nine studies involving 828 patients with unresectable HCC to compare the efficacy and safety of SBRT combined with TACE versus TACE (with or without concurrent systemic therapy). Our findings demonstrate that the addition of SBRT to TACE significantly improves both OS (HR = 0.48, 95% CI: 0.35-0.65) and PFS (HR = 0.45, 95% CI: 0.32-0.65) without a significant increase in the incidence of overall grade ≥ 3 treatment-related adverse events (RR = 1.27, 95% CI: 0.87-1.86). Notably, no cases of grade ≥3 radiation-induced liver injury were reported, underscoring the acceptable safety profile of this combined modality.

This study confirms that the combination of TACE and SBRT is significantly superior to TACE in improving survival outcomes. The underlying​ synergistic biological basis for this benefit likely stems from the complementary actions of the two modalities (16). TACE, by embolizing the tumor’s feeding arteries and locally releasing chemotherapeutic agents, effectively induces tumor ischemia and necrosis (33). However, the ensuing hypoxic environment may upregulate tumor cell radioresistance (34). Nevertheless,​ the subsequent delivery of SBRT, which precisely delivers ablative high radiation doses in few fractions, can effectively eradicate the residual, potentially more aggressive viable tumor cells remaining after TACE by overcoming hypoxia-induced radioresistance. This is partly because high-dose per fraction in SBRT reduces the oxygen enhancement ratio (35), thereby mitigating the impact of a hypoxic microenvironment while directly damaging tumor vasculature (27, 36). Furthermore, the chemotherapeutic agents retained within the hepatocellular carcinoma cells during embolization exert a radiosensitizing effect, accelerating tumor necrosis (37). For tumors with poor blood supply and suboptimal lipiodol emulsion deposition, radiotherapy can significantly enhance the tumor response rate (38). Therefore, the integration of these two modalities addresses the inherent limitations of either treatment monotherapy.

Additionally, administering radiotherapy after TACE can prolong the retention time of lipiodol and anticancer drugs within the tumor (39), thereby potentially obviating the need for repeated TACE sessions that could otherwise compromise liver function. This sequential treatment strategy is particularly suitable for lesions exhibiting an incomplete response to TACE (40, 41). Observational studies have demonstrated that this approach can enable patients to achieve favorable survival outcomes comparable to those with a complete response to TACE or those receiving other curative treatments (27, 38, 42). Moreover,​ Sahai et al. (43) also reported that multimodal therapy including SBRT may achieve downstaging and conversion surgery for advanced HCC with PVTT. The results of the present meta-analysis are consistent with these findings and, through the quantitative synthesis of multiple studies, provide higher-level evidence to support this conclusion. Furthermore, in the current era of systemic therapies, the TACE-SBRT combination may serve as a potent local-regional backbone that could potentially be integrated with emerging immunotherapies (44), as the ablative radiation dose may induce immunogenic cell death (45) and synergize with immune checkpoint inhibitors (46). Importantly, our meta-analysis reflects this contemporary paradigm by including studies that permitted concurrent systemic therapy (e.g., TKIs or ICIs) provided it was balanced between arms. The consistent survival benefit observed in the pre-specified subgroup analysis stratified by concurrent systemic therapy (present vs. absent) suggests that the therapeutic advantage of TACE-SBRT is maintained even in the context of modern systemic regimens, supporting its role as a versatile local-regional backbone.

When making clinical treatment decisions, treatment-related adverse events are a crucial consideration (47). This meta-analysis found no significant difference in the incidence of adverse events (CTCAE ≥ grade 3) between the TACE combined with SBRT group and the TACE group (RR = 1.27, 95% CI: 0.87–1.86, *P* = 0.21). Although the point estimate suggested a numerical increase of 27% in the risk of severe adverse events with combination therapy, the wide confidence interval crossing 1.0 indicated a lack of statistical significance, likely owing to limited event numbers and insufficient statistical power to detect a true difference. Nevertheless, these findings indicate that the combined modality of TACE and SBRT does not significantly increase the risk of severe adverse reactions and demonstrates a favorable safety profile. Notably, among the nine studies included in this analysis, there were no reports of grade ≥3 radiation-induced liver disease attributable to radiotherapy, nor were there any treatment-related deaths.

This safety profile can be attributed to several factors. On one hand, SBRT employs precise dose planning and image-guided techniques, which maximize the sparing of surrounding normal liver tissue and adjacent organs (48), thereby effectively reducing the probability of severe complications such as radiation-induced liver disease (49). On the other hand, in clinical practice, the safety of the combined regimen can be further optimized by judiciously adjusting parameters such as the dose of chemotherapeutic agents in TACE (50), as well as the fractionation scheme and total dose of SBRT (51), which helps mitigate​ the additive or synergistic toxicity that could theoretically arise from combining the two modalities (52). Therefore, the treatment strategy combining TACE with SBRT significantly improves patient survival outcomes without increasing severe safety risks, and patients generally exhibit good tolerance to this regimen (53). This characteristic aligns well with the treatment philosophy for patients with intermediate-to-advanced HCC, which emphasizes a dual focus on both efficacy and safety (54, 55).

Several limitations should be considered when interpreting the findings of this study. First, regarding study design and potential bias, the majority of included studies were non-randomized, which are subject to risks of unmeasured confounding (comorbidities, prior treatments, tumor grade, and the use of concurrent systemic therapies). Although addressed through subgroup and risk-of-bias analyses, residual confounding may weaken causal inferences. Second, the precision and generalizability of the estimates may be limited. Although 828 patients were pooled, many studies were single-center with modest sample sizes, which may limit the precision of the pooled effect estimates and their applicability to broader populations. Third, the scope of reported outcomes was incomplete. The analysis was restricted to OS, PFS, and severe adverse events. Other important endpoints, such as objective response rate, local control, quality of life, and detailed toxicity profiles (specific rates of radiation-induced liver disease), were not consistently reported and thus could not be synthesized. Finally, several important exploratory analyses were not feasible. Due to data limitations, we could not perform subgroup analyses based on key clinical factors like liver function (Child-Pugh class) or portal vein invasion, or investigate the impact of treatment parameters (SBRT dose, TACE regimen) on efficacy.

## Conclusion

In summary,​ based on current evidence, the combination of TACE with SBRT for unresectable hepatocellular carcinoma is associated with significant improvements in both overall survival and progression-free survival compared to TACE (with or without concurrent systemic therapy), without a significant increase in the risk of severe adverse events. This combined strategy offers an effective and well-tolerated treatment option for unresectable HCC. Future rigorously designed, large-scale RCTs are warranted to further validate these findings and to explore the optimal combined treatment regimen as well as the imaging or biological characteristics that identify the patient subgroups most likely to benefit.

## Data Availability

The original contributions presented in the study are included in the article/**Supplementary Material**. Further inquiries can be directed to the corresponding author.
