# Supplementary material for: Transcatheter arterial chemoembolization combined with stereotactic body radiation therapy versus transcatheter arterial chemoembolization for hepatocellular carcinoma: a systematic review and meta-analysis
Source: Front Oncol. 2026 Sep 17;16:1892798. doi: 10.3389/fonc.2026.1892798 (PMC13627024; doi:10.3389/fonc.2026.1892798)
Supplement: Supplementary file 1 [file Supplementaryfile1.docx]

**Transcatheter Arterial Chemoembolization Combined with Stereotactic Body Radiation Therapy versus Transcatheter Arterial Chemoembolization Alone for Hepatocellular Carcinoma: A Systematic Review and Meta-Analysis.**

Supplementary Table S1. PICO Framework.

Supplementary Table S2. Detailed search strategies for each database.

Supplementary Table S3. The detailed intervention content included in the study.

Supplementary Table S4. Risk of bias for the included studies, based on the ROBINS-I tool.

Supplementary Table S5. Certainty of evidence.

Supplementary Table S6. Assessment of methodological quality using AMSTAR 2 tool

Supplementary Figure S1. Risk of bias results for randomized controlled trials using the Cochrane RoB2 tool.

Supplementary Figure S2. Subgroup analysis forest plot for OS by study design (TACE+SBRT vs. TACE)

Supplementary Figure S3. Subgroup analysis forest plot for OS by adjustment status (TACE+SBRT vs. TACE).

Supplementary Figure S4. Subgroup analysis forest plot for OS by concurrent systemic therapy (TACE+SBRT vs. TACE).

Supplementary Figure S5. Subgroup analysis forest plot for PFS by study design (TACE+SBRT vs. TACE).

Supplementary Figure S6. Subgroup analysis forest plot for PFS by adjustment status (TACE+SBRT vs. TACE).

Supplementary Figure S7. Subgroup analysis forest plot for PFS by concurrent systemic therapy (TACE+SBRT vs. TACE).

Supplementary Figure S8. Sensitivity analysis forest plots (TACE+SBRT vs. TACE). (A) OS (HR); (B). PFS (HR); (C) Treatment-related adverse events (RR).

Supplementary Figure S9. Funnel plots and Egger’s test for publication bias assessment of primary endpoints (TACE+SBRT vs. TACE). (A) OS; (B) PFS; (C) Treatment-related adverse events.

Supplementary Table S1. PICO Framework.

| **Population (P)** | **Core Population**: Patients with a confirmed diagnosis of Hepatocellular Carcinoma (HCC) via histopathology or non-invasive imaging criteria (e.g., dynamic CT/MRI according to established guidelines like AASLD or EASL).  **Key Characteristics**:   - **Disease Stage**: Typically patients with Barcelona Clinic Liver Cancer (BCLC) stage B (intermediate) or selected stage C (advanced), who are unsuitable for curative therapies (e.g., resection, transplantation) but are eligible for TACE. - **Liver Function**: Patients with Child-Pugh class A or B liver function. - **Tumor Characteristics**: Patients with single or multiple nodules, with or without vascular invasion (the specific scope should be defined in the inclusion criteria). |
| --- | --- |
| **Interventions (I)** | **Core Regimen**: **TACE combined with SBRT**.  **Operational Definitions**:   - **TACE**: Includes conventional TACE (cTACE) or drug-eluting bead TACE (DEB-TACE). The timing (e.g., sequential) and role relative to SBRT should be specified. - **SBRT**: Delivery of stereotactic body radiation therapy, involving high-dose, hypofractionated, precisely targeted radiotherapy. Key parameters like prescription dose, fractionation schedule, and technique should be detailed. |
| **Control ©** | **Core Regimen**: **TACE alone**.  **Key Point**: The TACE methodology in the control group (e.g., chemotherapeutic agents, type of embolic material, treatment schedule) should be as consistent as possible with the TACE component in the combined treatment arm to ensure comparability. |
| **Outcomes (O)** | **Primary Outcome**:   - **Overall Survival (OS)**: Time from randomization or treatment initiation to death from any cause.   **Secondary Outcomes**:   - **Objective Response Rate (ORR)**: Rate of complete and partial responses according to the modified Response Evaluation Criteria in Solid Tumors (Mrecist) or similar criteria for HCC. - **Progression-Free Survival (PFS)**: Time from treatment initiation to disease progression or death from any cause. - **Local Control Rate**: Proportion of patients with controlled target lesions (absence of progression). - **Safety/Toxicity**: Incidence and severity of treatment-related adverse events (e.g., hepatic function impairment, radiation-induced liver disease, post-embolization syndrome). |
| **Study design (S)** | Randomized controlled trial (RCT) and non-randomized controlled trial |

Supplemental Table S2. Detailed search strategies for each database.

| Database | Search strategy |
| --- | --- |
| PubMed | ("Carcinoma, Hepatocellular"[Mesh] OR "hepatocellular carcinoma"[tiab] OR "HCC"[tiab] OR "hepatoma"[tiab] OR "liver cancer"[tiab] OR "primary liver cancer"[tiab])  AND  ("Chemoembolization, Therapeutic"[Mesh] OR "transcatheter arterial chemoembolization"[tiab] OR "TACE"[tiab] OR "transarterial chemoembolization"[tiab] OR "chemoembolization"[tiab])  AND  ("Radiosurgery"[Mesh] OR "Stereotactic Body Radiation Therapy"[Mesh] OR "stereotactic body radiation therapy"[tiab] OR "SBRT"[tiab] OR "stereotactic ablative radiotherapy"[tiab] OR "SABR"[tiab]) |
| Medline | #1. exp Carcinoma, Hepatocellular/  #2. (hepatocellular carcinoma or HCC or hepatoma or liver cancer or primary liver cancer).ti,ab,kf.  #3. 1 or 2  #4. exp Chemoembolization, Therapeutic/  #5. (transcatheter arterial chemoembolization or TACE or transarterial chemoembolization or chemoembolization).ti,ab,kf.  #6. 4 or 5  #7. exp Radiosurgery/  #8. exp Stereotactic Body Radiation Therapy/  #9. (stereotactic body radiation therapy or SBRT or stereotactic ablative radiotherapy or SABR).ti,ab,kf.  #10. 7 or 8 or 9  #11. 3 and 6 and 10 |
| Embase | #1. exp liver cell carcinoma/  #2. (hepatocellular carcinoma or HCC or hepatoma or liver cancer or primary liver cancer).ti,ab,kw.  #3. 1 or 2  #4. exp transarterial chemoembolization/  #5. (transcatheter arterial chemoembolization or TACE or transarterial chemoembolization or chemoembolization).ti,ab,kw.  #6. 4 or 5  #7. exp stereotactic body radiation therapy/  #8. (stereotactic body radiation therapy or SBRT or stereotactic ablative radiotherapy or SABR).ti,ab,kw.  #9. 7 or 8  #10. 3 and 6 and 9 |
| Cochrane Library | ("hepatocellular carcinoma" OR "HCC" OR "hepatoma" OR "liver cancer" OR "primary liver cancer")  AND  ("transcatheter arterial chemoembolization" OR "TACE" OR "transarterial chemoembolization" OR "chemoembolization")  AND  ("stereotactic body radiation therapy" OR "SBRT" OR "stereotactic ablative radiotherapy" OR "SABR") |
| Web of Science | (TI=("hepatocellular carcinoma" OR "HCC" OR "hepatoma" OR "liver cancer" OR "primary liver cancer") OR AB=("hepatocellular carcinoma" OR "HCC" OR "hepatoma" OR "liver cancer" OR "primary liver cancer"))  AND  (TI=("transcatheter arterial chemoembolization" OR "TACE" OR "transarterial chemoembolization" OR "chemoembolization") OR AB=("transcatheter arterial chemoembolization" OR "TACE" OR "transarterial chemoembolization" OR "chemoembolization"))  AND  (TI=("stereotactic body radiation therapy" OR "SBRT" OR "stereotactic ablative radiotherapy" OR "SABR") OR AB=("stereotactic body radiation therapy" OR "SBRT" OR "stereotactic ablative radiotherapy" OR "SABR")) |

Supplemental Table S3. The detailed intervention content included in the study.

| Study |  |  |
| --- | --- | --- |
| Honda (2012) | SBRT | **Timing:** Performed 1–2 months (median: 1 month) after TACE.  **Dose/Fractionation:** The primary regimen was 48 Gy delivered in 4 fractions (26 cases). For tumors near critical organs (e.g., inferior vena cava, gallbladder), the dose was 60 Gy delivered in 8 fractions (4 cases) to reduce the dose per fraction. Treatment was completed within 4–10 days.  **Immobilization & Respiratory Management:** Patients were immobilized using a vacuum cushion. Breath-hold was performed at the end-expiration phase using a self-control device (Abches).  **Target Volume Delineation:**  • GTV: Defined as the tumor volume, represented by the remains of lipiodol from TACE and early enhancement on dynamic CT.  • CTV: A 3 mm margin was added to the GTV.  • PTV: A 5–8 mm margin was added to the CTV to account for respiratory motion and setup error.  **Radiation Technique:** Treatment was delivered using 6–10 MV photons from a linear accelerator via eight non-coplanar ports (including 4 coplanar and 4 non-coplanar beams), avoiding the intestine, gallbladder, esophagus, and spine as much as possible. |
|  | TACE | **Access Route:** Performed via the femoral artery using the Seldinger technique under local anesthesia.  Chemotherapeutic Agents: Cisplatin (concentration: 10 mg/mL, dose: 7–70 mg/body) or Miriplatin (concentration: 20 mg/mL, dose: 20–80 mg/body) mixed with iodized oil. Cisplatin was used before January 2010 and was subsequently replaced by Miriplatin.  **Dosing:** The selected dose was based on tumor size and the extent of liver damage.  **Endpoint:** Injection was discontinued upon full accumulation of iodized oil in the tumor vessels.  **Post-procedure:** A gelatin sponge was used for embolization after TACE. |
| Paik (2016) | SBRT | **Timing:** SABR was performed for patients with an incomplete response to TACE.  **Dose/Fractionation:** A total dose of 40-60 Gy (median 56 Gy) was prescribed in 3-5 fractions. Doses were determined per institutional protocols, with 60 Gy in 3 fractions as a starting point, then reduced by 0.5 or 1 Gy per fraction until normal tissue constraints were met.  **Immobilization & Respiratory Management:** Simulation CT was performed with abdominal compression. Both slow and helical CT images were obtained. The slow CT images captured the respiratory motion of the target.  Target Volume Delineation:  • GTV: Defined on the slow CT set as the viable tumor with contrast uptake, including embolization material.  • ITV: The GTV defined on the slow CT, which included respiratory motion, was used directly as the Internal Target Volume (ITV).  • PTV: A margin of 0-4 mm was added to the ITV to create the Planning Target Volume.  **Radiation Technique:**  • Systems: Treatment was delivered using either a CyberKnife or a RapidArc system.  • Image Guidance:    - CyberKnife: Used gold fiducials inserted near the spine closest to the lesion for tracking.    - RapidArc: Used gold fiducials inserted in the liver near the tumor or lipiodol uptake as markers for cone-beam CT guidance.  **Dose Prescription:** The dose was prescribed to the 70-80% isodose line, which was required to cover at least 97% of the PTV. |
|  | TACE | **Procedure & Embolic Agent:** TACE was performed with an infusion of a mixture of lipiodol and doxorubicin.  **Response Assessment & Treatment Strategy:** Tumor response was evaluated via CT one month after TACE. If a complete response was achieved, no additional treatment was given, and the patient was followed up. In cases of incomplete response, additional TACE or a change in treatment strategy (e.g., SABR) was conducted.  **Endpoint (Definition of Incomplete Response):** Incomplete response was defined as disease progression or incomplete tumor filling by the lipiodol-doxorubicin mixture on CT images. Disease progression included residual viable tumors, progression of existing lesions, or development of new lesions. |
| Jun 2018 | SBRT | **Timing:** SBRT was performed for viable tumors that showed an incomplete response after the first TACE based on follow-up CT.  **Dose and Fractionation:** A total dose of 40-60 Gy (median, 55 Gy) was administered to the PTV in three to five fractions over consecutive days or twice a week.  **Patient Immobilization and Respiratory Management:** Immobilization: Patients were immobilized in a supine position with arms above their head. Gross Tumor Volume (GTV) was measured based on CT images at the end-expiratory phase.  **Target Volume Delineation:**  • GTV: Defined based on end-expiratory phase CT images fused with multi-phase MR images.  • ITV: Set as the volume encompassing movement within the gating phase (30-70%) from the GTV.  • PTV: Defined as the volume with a 5 mm margin added to the ITV.  **Irradiation Technique:** The study utilized four different radiotherapy systems across the participating centers:  CyberKnife Radiosurgery System (Accuray).  Novalis TX (Varian Medical Systems and BrainLab).  TomoTherapy device.  TrueBeam medical linear accelerator (Varian Medical Systems). |
|  | TACE | **Procedure Access:** Performed via the common femoral artery using an angiographic catheter.  **Anticancer Drugs:** A mixture of intra-arterial adriamycin (doxorubicin, 50 mg/m²) and lipiodol (5 to 10 mL) was used.  Protocol Selection: TACE served as the initial treatment for eligible patients (tumor ≤5 cm, ≤3 lesions, ineligible for resection or local ablation).  **Operational Endpoint:** The feeder vessels of the hepatic segments containing the tumor were selected.  Post-procedure Management: Gelfoam (gelatin sponge) embolization was performed following the infusion of the chemotherapeutic mixture. |
| Wong 2019 | SBRT | **Timing:** SBRT was delivered at least 1 week after TACE, commencing once the patient's general condition and biochemical parameters recovered to pretreatment levels.  **Dose and Fractionation:** Two primary regimens were used:  For patients with tumor size ≤10cm and ECOG 1-2: 5-8.5 Gy per fraction for 6 fractions.  For patients with tumor >10 cm and borderline liver function: 4 Gy per fraction for 6-10 fractions.  The total dose ranged from 30 Gy (5Gy x 6) to 40 Gy (4Gy x 10).  **Patient Immobilization and Respiratory Management:**  Immobilization: Patients were immobilized with a vaclock plus, an in-house body frame with an abdominal compressor.  Respiratory Management: Four-dimensional CT (4DCT) was acquired using a bellows-belt. The 4DCT dataset was sorted into ten respiratory phases, and the mid-ventilation phase was chosen as the planning CT.  **Target Volume Delineation:**  GTV: Defined as the HCC focus that demonstrated either arterial enhancement or washout at the portovenous/delayed phase on triphasic contrast CT.  CTV: Defined as the GTV with an expansion to include the lipiodol-stained area to cover potential microscopic residual disease.  PTV: Generated from the liver and tumor motions using the Van Herk margin recipe.  **Irradiation Technique:** Treatment was planned and delivered using either 6 or 10 MV photons based on tumor size and location. The goal was to use the highest allowable dose while respecting normal tissue constraints for organs at risk (OARs) and the liver (mean liver dose and V30Gy3). |
|  | TACE | **Procedure Access:** Celiac and superior mesenteric arterial and portovenogram were done to define arterial anatomy. The right or left hepatic artery was superselectively catheterized.  **Anticancer Drugs:** A mixture of cisplatin (1mg/ml) with lipiodol in a volume ratio of 1:1 was prepared. A maximum of 60 ml (equivalent to 30 mg cisplatin) of the mixture was used.  **Protocol Selection:** TACE was given twice at an 8-week interval.  Operational Endpoint: The mixture was injected under fluoroscopic monitoring. The amount used depended on tumor size, number, and arterial blood flow.  **Post-procedure Management:** This was followed by embolization with gelfoam pellets of 1 mm diameter mixed with 40 mg gentamicin. |
| Comito | SBRT | **Treatment Timing:** SBRT was delivered 6-8 weeks after the first TAE/TACE, following CT-based detection of residual disease.  Dose and Fractionation: SBRT was delivered in 3 to 6 fractions. The dose schedule was adapted individually. The median delivered dose was 60 Gy (range 30-75 Gy) in 6 fractions (range 3-10).  **Biological Effective Dose:** 88 Gy_10_ (60-96 Gy_10_)  **Patient Immobilization and Respiratory Management:**  Immobilization: Not specified in the provided text. Tumor motion was managed through 4D CT and abdominal compression.  Target Volume Delineation:  GTV: Delineated on pretreatment imaging (4D simulation CT). Image fusion with MRI and/or choline PET was used when available.  CTV: Corresponded to the GTV.  PTV: Corresponded to the CTV plus a 7-10 mm isotropic expansion.  **Irradiation Technique:** The study does not specify the exact radiation delivery technology (e.g., linear accelerator type). |
|  | TACE | **Procedure Access:** The superselective catheterization of feeding vessels was obtained using microcatheters (Renegade High Flow) to perform subsegmental embolizations.  Anticancer Drugs & Embolic Agents:  TAE: Embolization was performed using calibrated microparticles (40±10 and/or 100±25 μm in diameter) with a hydrogel core and a Polyzene-F coating (Embozene Color-Advanced Microspheres).  TACE: A mixture of 5 mL of iodized oil (Lipiodol) and 50 mg of epirubicin dissolved in 5 mL of non-ionic contrast media was used, followed by particle administration (same microparticles as TAE).  **Protocol Selection:** Patients randomized to this arm received the procedure (TAE or conventional TACE) according to the first embolization treatment they received prior to randomization.  **Operational Endpoint:** The procedure was considered completed only when vascular shutdown was confirmed and no feeding vessels to the target tumor were detected at final angiography. For TAE, 40 μm microspheres were injected until blood flow interruption or after administering 4 mL; 100 μm particles were used if residual enhancement persisted.  **Post-procedure Management:** Does not specify additional management post-embolization. |
| Xiang 2022 | SBRT | **Treatment Timing:** SBRT was administered after patients were diagnosed as refractory to TACE treatment. Patients were switched from TACE to receive SBRT combined with PD-1 inhibitors.  Dose and Fractionation: SBRT was delivered as a total dose of 24-45 Gy in 3-5 fractions.  **Patient Immobilization and Respiratory Management: Immobilization / Fiducial Placement:** Patients receiving SBRT were first implanted with at least 3 gold fiducials inside or adjacent to the tumour under CT guidance. A localization CT simulation was performed after the fiducials were stable.  Respiratory Management: The study does not specify the respiratory motion management technique used (e.g., 4DCT, abdominal compression).  **Target Volume Delineation:**  GTV: Defined as the radiologically evident gross disease.  PTV: Created by adding a 2-5 mm marginal expansion to the GTV.  Irradiation Technique: Treatment was delivered using the CyberKnife® system (Accuray Cyberknife, VSI). Treatment plans were developed respecting normal tissue dose constraints based on AAPM Task Group 101 guidelines.  3. PD-1 Inhibitors (Combined Therapy)  **Drugs and Dosing:** All patients received PD-1 inhibitors after TACE refractoriness. The inhibitors used were toripalimab (72.4% of patients) at 3 mg/kg every 2 weeks or sintilimab (27.6%) at 200 mg every 3 weeks. They were administered intravenously and continued until disease progression or intolerable toxicity. |
|  | TACE | **Procedure Access:** TACE was performed using Seldinger's technique. The tumour-feeding artery was first identified by angiography, and cannulation of the hepatic artery was achieved.  **Anticancer Drugs:** A mixture of doxorubicin hydrochloride, pirarubicin and lipiodol was injected through the catheter. The specific doses for each drug are not detailed in the study.  **Protocol Selection:** TACE was the standard initial treatment for patients with intermediate-stage (BCLC B) HCC. The diagnosis of TACE refractoriness was based on the definition by the Japan Society of Hepatology, specifically an insufficient response of the treated tumour after two TACE procedures.  **Operational Endpoint:** The procedure involved the injection of the chemotherapeutic mixture after cannulation of the tumour-feeding artery.  **Post-procedure Management:** Does not specify the use of additional embolic agents (like gelatin sponge) following the chemoinfusion. |
| Saad 2024 | SBRT | **Treatment Timing:** SBRT was started 1 month after the end of TACE.  **Dose and Fractionation:** A total dose of 40 Gy was prescribed in 5 consecutive daily fractions over 1 week (8 Gy per fraction). **The Biological Effective Dose (BED) was 88 Gy.**  **Patient Immobilization and Respiratory Management:** Patients were immobilized in the supine position using a wing board. Each patient underwent a CT scan assisted by an inspiratory breath-hold technique.  **Target Volume Delineation:**  GTV: Delineated and guided by available diagnostic imaging (MRI, triphasic CT, or PET scan) and the lipiodol deposit in the chemo-embolized lesion.  CTV: No Clinical Target Volume (CTV) was created.  PTV: Created by adding a 0.5 cm margin around the GTV.  **Irradiation Technique:** Treatment was planned using Volumetric Modulated Arc Therapy (VMAT) on the Eclipse TPS. Plans respected organ-at-risk dose constraints. Treatment was delivered on a "UNIQUE" Varian linear accelerator. Cone beam computed tomography (CBCT) was performed daily prior to each session for patient positioning. |
|  | TACE | **Procedure Access:** Using the Seldinger technique, a catheter (4F to 5F) was inserted into the abdominal aorta through the right femoral artery. Selective arteriography of the hepatic artery was performed.  **Anticancer Drugs:** An emulsion containing lipiodol (2-15 ml) and cisplatin (20-100 mg) was administered.  **Protocol Selection:** TACE was the standard treatment. In the study, patients in Arm B received TACE followed by SBRT.  **Operational Endpoint:** The chemotherapeutic emulsion was administered into the tumor-feeding vessels identified by angiography.  **Post-procedure Management:** The study does not specify the use of additional embolic agents (like gelatin sponge) following the chemoinfusion. |
| Duan 2024 | SBRT | **The study did not report on the detailed intervention methods.** |
|  | TACE | **The study did not report on the detailed intervention methods.** |
| Chen 2025 | SBRT | **Treatment Timing:** SBRT was administered after TACE. The specific interval is not stated, but follow-up for response assessment was conducted one month post-treatment.  **Dose and Fractionation:** The prescribed regimen was 40 Gy delivered in 5 daily fractions over one week (8 Gy per fraction). **The Biological Effective Dose (BED) was 88 Gy.**  Patient Immobilization and Respiratory Management:  **Immobilization:** Patients were immobilized in a supine position using a wing board.  **Respiratory Management:** A contrast-enhanced CT simulation was performed during breath-hold at inspiration.  **Target Volume Delineation:**  GTV: Defined using multi-modality imaging (MRI, triphasic CT, or PET scan), with TACE-induced lipiodol retention serving as an additional guide.  CTV: No Clinical Target Volume (CTV) was created.  PTV: Created by adding a 0.5 cm margin to the GTV.  **Irradiation Technique:**  Planning & Delivery: Treatment was planned using volumetric modulated arc therapy (VMAT) with dose distributions optimized via dose-volume histograms. All treatments were delivered using a Varian "UNIQUE" linear accelerator.  Image Guidance: Cone-beam CT (CBCT) was used daily for precise patient positioning. |
|  | TACE | **Procedure Access:** The TACE procedure employed the Seldinger technique. A French catheter (4-5F) was inserted via the right femoral artery and advanced to the abdominal aorta.  **Anticancer Drugs:** An emulsion containing lipiodol and cisplatin was injected. The specific dosage of cisplatin is adjusted according to the size of the tumor.  **Protocol Selection:** TACE was the initial treatment. In the study, patients in the experimental group (Group B) received sequential TACE followed by SBRT.  **Operational Endpoint:** Selective hepatic arteriography was used for tumor visualization. The chemotherapeutic emulsion was administered into the targeted vessels.  **Post-procedure Management:** The study does not specify the use of additional embolic agents (like gelatin sponge) following the chemoinfusion in the methods section. |

| Supplementary Table S4. Risk of bias for the included studies, based on the ROBINS-I tool. | | | | | | | | | | |
| --- | --- | --- | --- | --- | --- | --- | --- | --- | --- | --- |
| Author | Year | Type of bias | | | | | | | | Overall rating |
|  |  | Confounding | Selection of participants | Measurement classification of interventions | | Deviations from intended interventions | Missing data | Measurement of the outcomes | Selective reporting of the results |  |
| Honda | 2013 | Moderate | Moderate | Low | Low | | Low | Low | Low | Moderate |
| Paik | 2016 | Moderate | Moderate | Low | Low | | Low | Low | Low | Moderate |
| Jun | 2018 | Low | Low | Low | Low | | Low | Low | Low | Low |
| Wong | 2019 | Low | Low | Low | Low | | Low | Low | Low | Low |
| Xiang | 2022 | Moderate | Moderate | Low | Low | | Low | Low | Low | Moderate |
| Chen | 2025 | Moderate | Moderate | Low | Low | | Low | Low | Low | Moderate |

Supplementary Table S5. Certainty of evidence.

| Outcomes | **Anticipated absolute effects^*^** (95% CI) | | Relative effect (95% CI) | № of participants (studies) | Certainty of the evidence (GRADE) |
| --- | --- | --- | --- | --- | --- |
|  | **Risk with TACE** | **Risk with SBRT + TACE** |  |  |  |
| OS | **Low** | | **HR 0.47** (0.36 to 0.62) [OS] | 698 (7 non-randomised studies) | ⨁⨁⨁◯ Moderate |
|  | 0 per 1,000 | **0 per 1,000** (0 to 0) |  |  |  |
| DFS | **Low** | | **HR 0.50** (0.40 to 0.62) [DFS] | 721 ( 8 non-randomised studies) | ⨁⨁⨁◯ Moderate |
|  | 1 per 1,000 | **1 per 1,000** (1 to 1) |  |  |  |
| Treatment-Related Adverse Events | 123 per 1,000 | **156 per 1,000** (107 to 228) | **RR 1.27** (0.87 to 1.86) | 721 (8 non-randomised studies) | ⨁⨁⨁◯ Moderate |
| ***The risk in the intervention group** (and its 95% confidence interval) is based on the assumed risk in the comparison group and the **relative effect** of the intervention (and its 95% CI).  **CI:** confidence interval; **HR:** hazard ratio; **RR:** risk ratio | | | | | |
| **GRADE Working Group grades of evidence** **High certainty:** we are very confident that the true effect lies close to that of the estimate of the effect. **Moderate certainty:** we are moderately confident in the effect estimate: the true effect is likely to be close to the estimate of the effect, but there is a possibility that it is substantially different. **Low certainty:** our confidence in the effect estimate is limited: the true effect may be substantially different from the estimate of the effect. **Very low certainty:** we have very little confidence in the effect estimate: the true effect is likely to be substantially different from the estimate of effect. | | | | | |

Supplemental Table S6. Assessment of methodological quality using AMSTAR 2 tool

| Item 1 | Item 2 | Item 3 | Item 4 | Item 5 | Item 6 | Item 7 | Item 8 | Item 9 | Item 10 | Item 11 | Item 12 | Item 13 | Item 14 | Item 15 | Item 16 | Overall quality |
| --- | --- | --- | --- | --- | --- | --- | --- | --- | --- | --- | --- | --- | --- | --- | --- | --- |
| Y | Y | Y | Y | Y | Y | PY | Y | Y | PY | Y | Y | Y | Y | Y | Y | High |

Note:

Item 1: Did the research questions and inclusion criteria for the review include the components of PICO?

Item 2: Did the report of the review contain an explicit statement that the review methods were established prior to the conduct of the review and did the report justify any significant deviations from the protocol?

Item 3: Did the review authors explain their selection of the study designs for inclusion in the review?

Item 4: Did the review authors use a comprehensive literature search strategy?

Item 5: Did the review authors perform study selection in duplicate?

Item 6: Did the review authors perform data extraction in duplicate?

Item 7: Did the review authors provide a list of excluded studies and justify the exclusions?

Item 8: Did the review authors describe the included studies in adequate detail?

Item 9: Did the review authors use a satisfactory technique for assessing the risk of bias (RoB) in individual studies that were included in the review?

Item 10: Did the review authors report on the sources of funding for the studies included in the review?

Item 11: If meta-analysis was performed did the review authors use appropriate methods for statistical combination of results?

Item 12: If meta-analysis was performed, did the review authors assess the potential impact of RoB in individual studies on the results of the meta-analysis or other evidence synthesis?

Item 13: Did the review authors account for RoB in individual studies when interpreting/ discussing the results of the review?

Item 14: Did the review authors provide a satisfactory explanation for, and discussion of, any heterogeneity observed in the results of the review?

Item 15: If they performed quantitative synthesis did the review authors carry out an adequate investigation of publication bias (small study bias) and discuss its likely impact on the results of the review?

Item 16: Did the review authors report any potential sources of conflict of interest, including any funding they received for conducting the review.

Supplementary Figure S1. Risk of bias results for randomized controlled trials using the Cochrane RoB2 tool.

Supplementary Figure S2. Subgroup analysis forest plot for OS by study design (TACE+SBRT vs. TACE)

Supplementary Figure S3. Subgroup analysis forest plot for OS by adjustment status (TACE+SBRT vs. TACE).

Supplementary Figure S4. Subgroup analysis forest plot for OS by concurrent systemic therapy (TACE+SBRT vs. TACE).

Supplementary Figure S5. Subgroup analysis forest plot for PFS by study design (TACE+SBRT vs. TACE).

Supplementary Figure S6. Subgroup analysis forest plot for PFS by adjustment status (TACE+SBRT vs. TACE).

Supplementary Figure S7. Subgroup analysis forest plot for PFS by concurrent systemic therapy (TACE+SBRT vs. TACE).

Supplementary Figure S8. Sensitivity analysis forest plots (TACE+SBRT vs. TACE). (A) OS (HR); (B). PFS (HR); (C) Treatment-related adverse events (RR).

Supplementary Figure S9. Funnel plots and Egger’s test for publication bias assessment of primary endpoints (TACE+SBRT vs. TACE). (A) OS; (B) PFS; (C) Treatment-related adverse events.
